# Anti-Inflammatory Effect of Streptochlorin via TRIF-Dependent Signaling Pathways in Cellular and Mouse Models

**DOI:** 10.3390/ijms16046902

**Published:** 2015-03-26

**Authors:** Do-Wan Shim, Hee Jae Shin, Ji-Won Han, Woo-Young Shin, Xiao Sun, Eun-Jeong Shim, Tack-Joong Kim, Tae-Bong Kang, Kwang-Ho Lee

**Affiliations:** 1 Department of Biotechnology, College of Biomedical and Health Science, Konkuk University, Chungju 380-150, Korea; E-Mails: simdw871113@gmail.com (D.-W.S.); frbgf@naver.com (J.-W.H.); wooyoung031@naver.com (W.-Y.S.); sunxiao0507@naver.com (X.S.); sim1023@hanmail.net (E.-J.S.); kangtbko@gmail.com (T.-B.K.); 2 Marine Natural Products Chemistry Laboratory, Korea Institute of Ocean Science & Technology, 787 Haeanro, Ansan 426-744, Korea; E-Mail: shinhj@kiost.ac; 3 Division of Biological Science and Technology, Yonsei University, Wonju 222-710, Korea; E-Mail: ktj@yonsei.ac.kr

**Keywords:** streptochlorin, LPS, anti-inflammation, TRIF, ALI

## Abstract

Streptochlorin, a small compound derived from marine actinomycete, has been shown to have anti-angiogenic, anti-tumor, and anti-allergic activities. However, the anti-inflammatory effects and underlying mechanisms have not yet been reported. In the present study, we investigated the effect of streptochlorin on lipopolysaccharide (LPS)-induced inflammatory responses *in vitro* and *in vivo*. Streptochlorin attenuated the production of proinflammatory mediators such as nitric oxide, cyclooxygenase-2, pro-interleukin (IL)-1β, and IL-6 in LPS-stimulated RAW264.7 cells through inhibition of the Toll/interleukin-1 receptor (TIR)-domain-containing adapter-inducing interferon-β (TRIF)-dependent signaling pathway. Furthermore, streptochlorin suppressed the infiltration of immune cells such as neutrophils into the lung and proinflammatory cytokine production such as IL-6 and TNF-α in broncho-alveolar lavage fluid (BALF) in the LPS-induced acute lung injury (ALI) mouse model. Streptochlorin has potent anti-inflammatory effects through regulating TRIF-dependent signaling pathways, suggesting that streptochlorin may provide a valuable therapeutic strategy in treating various inflammatory diseases.

## 1. Introduction

Inflammation is a host defense mechanism against pathogens, but chronic inflammation is related to many diseases such as inflammatory bowel disease and rheumatoid arthritis [1]. Macrophages play an important role in inflammation, and are regarded as a suitable *in vitro* model for evaluating the potency of anti-inflammatory drugs and exploring the mechanism of their action [2]. Lipopolysaccharide (LPS) is recognized by toll-like receptor 4 (TLR4) and leads to the activation of two different signal pathways, MyD88- and Toll/interleukin-1 receptor (TIR)-domain-containing adapter-inducing interferon-β (TRIF)-dependent pathways [3]. The TRIF-dependent signaling pathway induces the activation of the interferon regulatory factor (IRF3), the transcriptional regulator, the late-phase activation of NF-κB, and mitogen-activated protein kinase (MAPK) [4,5]. The TRIF-dependent signaling pathway induces inflammatory cytokines and Type I interferons (IFNs) and IFN-inducible genes [6,7].

Acute lung injury (ALI) is induced by many extreme conditions and characterized by an increased permeability of the alveolar-capillary barrier, resulting in lung edema with protein-rich fluid, consequently leading to impairment of arterial oxygenation [8]. LPS inhalation mimics human gram-negative ALI, inducing neutrophil recruitment, pulmonary edema, and finally impairment of gas exchange [9].

Streptochlorin, a yellowish amorphous compound isolated from *Streptomyces* sp., possesses selective cytotoxicity against several cancer cell lines [10,11]. We have previously reported that streptochlorin has anti-allergic activity in RBL-2H3 cells [12]. In this study, we report the anti-inflammatory effects of streptochlorin and underlying mechanisms involved in both cellular and animal models.

## 2. Results and Discussion

### 2.1. Streptochlorin Inhibited the Production of Proinflammatory Mediators in Lipopolysaccharide (LPS)-Stimulated RAW264.7 Cells

**Figure 1:**
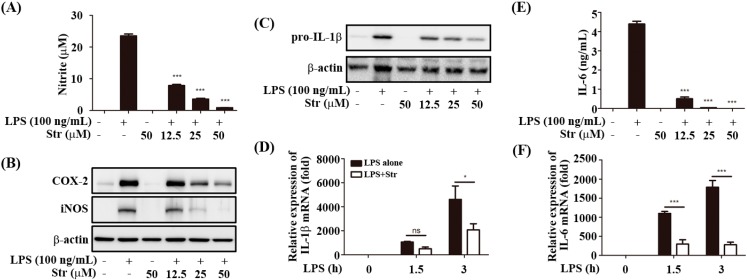

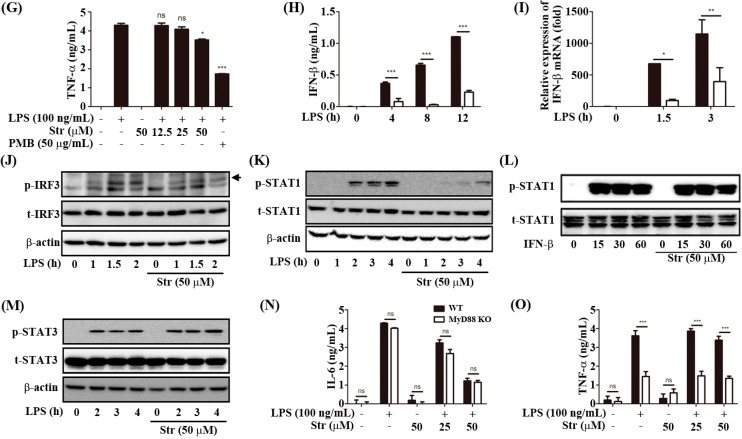
Effect of streptochlorin on the production of inflammatory mediators in lipopolysaccharide (LPS)-stimulated RAW264.7 cells. RAW264.7 cells were pretreated with streptochlorin (Str) at indicated concentration for 1 h and then treated with LPS (100 ng/mL). After incubation for 24 h, (**A**) the nitrite level in the culture supernatant was measured using nitric oxide (NO) assay; (**B**) The level of inducible nitric oxide synthase (iNOS) and cyclooxygenase-2 (COX-2) after 18 h of incubation was determined by western blot; Both protein (**C**) and mRNA level (**D**) of pro-IL-1β after 4 h and indicated time incubation, IL-6 (**E**,**F**) after 16 h and indicated time incubation, tumor necrosis factor α (TNF-α) (**G**) after 4 h of incubation, and IFN-β (**H**,**I**) after indicated time incubation was determined by western blot analysis, enzyme-linked immunosorbent assay (ELISA), and real-time polymerase chain reaction (PCR). After indicated time of incubation, phosphorylation of IRF3 (**J**), signal transduction and activation of transcription 1 (STAT1) (**K**), and STAT3 (**M**) was determined by western blot analysis. RAW264.7 cells were pretreated with streptochlorin at 50 μM for 1 h and then treated with IFN-β (100 U/mL); (**L**) After indicated incubation time, phosphorylation of STAT1 was determined by western blot analysis. Wild type (WT) and MyD88 knockout (Myd88 KO) mice-originated peritoneal macrophages were pretreated with LPS. After incubation for 16 and 3 h, the protein level of IL-6 (**N**) and TNF-α (**O**) was determined by ELISA. The results are expressed as mean ± SEM (*n* = 3). * *p* < 0.05, ** *p* < 0.01, and *** *p* < 0.001 compared with LPS-treated cells. n.s.: none significant.

Streptochlorin at concentrations up to 100 μM did not affect the viability of RAW264.7 cells. Therefore, the maximum concentration of streptochlorin was used at 50 μM for the following analyses. As shown in Figure 1A,B, streptochlorin significantly inhibited nitric oxide (NO) production through decreased inducible nitric oxide synthase (iNOS) protein expression [13] and also inhibited cyclooxygenase-2 (COX-2) in RAW264.7 cells. Streptochlorin also inhibited both the protein and mRNA level of pro-IL-1β and IL-6 (Figure 1C–F). However, streptochlorin slightly inhibited LPS-induced tumor necrosis factor α (TNF-α) secretion (Figure 1G). Interestingly, streptochlorin significantly inhibited both the protein and mRNA level of IFN-β in RAW264.7 cells (Figure 1H,I). The TRIF-dependent signals lead to activation of IRF3 and IFN-β expression [14]. IFN-β in turn induces activation of signal transduction and activation of transcription 1 (STAT1) signaling, which mediates the expression of an inducible gene such as iNOS [15] and induces IL-6 and pro-IL-1β [16]. Streptochlorin significantly inhibited LPS-induced IRF3 phosphorylation in RAW264.7 cells (Figure 1J). Therefore, we examined the effect of streptochlorin on the LPS-induced STAT1 activation in RAW264.7 cells. Streptochlorin significantly inhibited LPS-induced STAT1 activation in RAW264.7 cells (Figure 1K). However, STAT1 phosphorylation was not changed by streptochlorin in IFN-β-primed cells (Figure 1L), indicating that downstream signals from IFN-β receptor were not affected by streptochlorin. In our previous study, streptochlorin inhibited FcεRI-mediated tyrosine kinase such as Lyn, Fyn, and Syk, in IgE/DNP-HSA-stimulated RBL-2H3 cells [12]. The Jak/STAT pathway is well known to be tyrosine kinase [17]. As seen in Figure 1K, streptochlorin strongly inhibited phosphorylation of STAT1 tyrosine residue; therefore we examined the effect of streptochlorin on the phosphorylation of STAT3 tyrosine residue in LPS-stimulated RAW264.7 cells. Streptochlorin did not affect LPS-induced STAT3 tyrosine phosphorylation in RAW264.7 cells (Figure 1M), indicating that streptochlorin did not inhibit LPS-mediated tyrosine phosphorylation in RAW264.7 cells. To verify the role of streptochlorin on the LPS-induced TRIF-dependent signal pathway, we examined the effect of streptochlorin on LPS-stimulated peritoneal macrophages from MyD88 knockout mice. As shown in Figure 1N, streptochlorin significantly inhibited IL-6 production both LPS-stimulated wild type and MyD88 knockout peritoneal macrophages. However, streptochlorin did not inhibit TNF-α production neither wild type nor MyD88 knockout peritoneal macrophages (Figure 1O). These data suggested that streptochlorin suppressed LPS-mediated inflammatory responses mainly through TRIF-dependent signal pathway. In addition, streptochlorin did not show any inhibitory role in LPS-induced MAPK signaling in RAW264.7 cells and matured IL-1β secretion induced by NLRP3 inflammasome activation in bone marrow-derived macrophages. In our previous paper, we have reported that streptochlorin suppressed the MAPK signaling [12]. It is remained to be clarified how streptochlorin affects to MAPK with different manner. Taken together, our data indicated that streptochlorin inhibited the LPS-induced inflammatory responses by the inhibition of TRIF-dependent signals.

### 2.2. Streptochlorin Ameliorated LPS-Induced ALI in Mice

**Figure 2:**
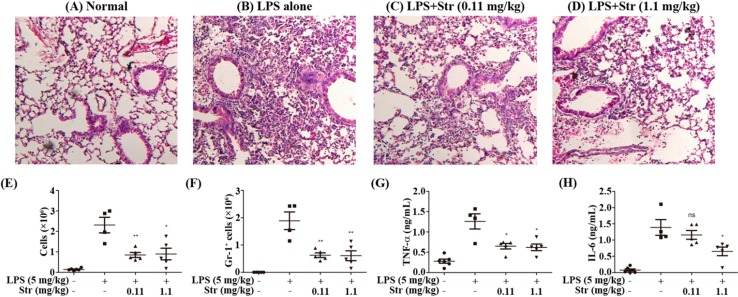
Effects of streptochlorin on LPS-induced ALI mouse model. BALB/c mice were given an intraperitoneal injection of streptochlorin successively 12 h and 2 h before being treated with LPS. (**A**–**D**) Histological examination of lung tissues was performed 24 h after the LPS challenge; Broncho-alveolar lavage fluid (BALF) was collected at 24 h after the LPS challenge to measure the total number of cells (**E**) and GR-1^+^ cells (**F**) were measured by flow cytometry; The concentrations of TNF-α (**G**) and IL-6 (**H**) in BALF after LPS prime alone or with indicated amounts of streptochlorin were measured by ELISA. The results are expressed as mean ± SEM. * *p* < 0.05, ** *p* < 0.01, and compared with LPS-treated group.

Endotoxin or LPS derived from Gram-negative bacteria has been well recognized in the pathogenesis of ALI [18]. Therefore, to support our *in vitro* data, we extended our work to the *in vivo* mouse model. The effects of streptochlorin on cell infiltration into lung and proinflammatory cytokine secretion in BALF were monitored by lung tissue H&E staining, cell counting, and enzyme-linked immunosorbent assay (ELISA). As shown in Figure 2A, the infiltration of inflammatory cells was not detected in the alveolar spaces of mice in the normal group. In contrast, large numbers of inflammatory cells were recruited into the alveolar spaces after LPS treatment (Figure 2B). However, treatment with streptochlorin at the indicated doses markedly attenuated inflammatory cell infiltration (Figure 2C,D). We also measured the total cell number and neutrophils in BALF by hemacytometer and FACS. The total inflammatory cells and neutrophils in BALF after 24 h treatment with intranasal instillation of LPS were significantly elevated in LPS-induced mice. But both the total number of cells and the number of neutrophils in the lung tissue were significantly reduced in the mice treated with streptochlorin (Figure 2E,F). Furthermore, inflammatory cytokines such as TNF-α and IL-6 in the BALF were also decreased by streptochlorin treatment (Figure 2G,H). No major side-effects were observed with tested doses of streptochlorin treatment including the body weight loss. These results indicate that streptochlorin has a protective effect on LPS-induced ALI by reducing inflammatory cells such as neutrophil recruitment and proinflammatory cytokine production at the inflammation site.

## 3. Experimental Section

### 3.1. Reagents and Antibodies

The preparation process of streptochlorin has been described previously [12]. Penicillin, streptomycin, Dulbecco’s modified Eagle’s medium (DMEM), and fetal bovine serum (FBS) were purchased from Gibco (Grans Island, NY, USA). The IFN-β ELISA kit was purchased from Biolegend (San Diego, CA, USA). TNF-α, IL-6 ELISA Set (BD OptEIA TMSet) and TMB Substrate Reagent Set were purchased from BD Biosciences (Franklin Lakes, SD, USA). Antibodies against iNOS, COX-2, IL-1β, and β-actin were purchased from Santa Cruz Biotechnology (Santa Cruz, CA, USA). Antibodies against phosphor and the total form of IRF3 (S396) and STAT1 (Y701) and STAT3 (Y705) were purchased from Cell Signaling Technology, Inc. (Beverly, MA, USA). Recombinant mouse IFN-β was purchased from PBL Interferon Source (Piscataway, NJ, USA). Western blot chemiluminescence reagent kits (Super Signal West Pico Stable Peroxide and Super Signal West Pico Luminol/Enhancer solutions) were purchased from Pierce Chemical (Rockford, IL, USA). Polyvinylidene fluoride (PVDF) membrane was purchased from Millipore Corporation (Bedford, MA, USA). LPS (*E. coli* 026:B6) and other chemicals were purchased from Sigma–Aldrich (St. Louis, MO, USA). All other chemicals used in our study were of the highest quality.

### 3.2. Cell Culture

Murine macrophage cell line, RAW 264.7, was obtained from the American Type Culture Collection (ATCC, Rockville, MD, USA) and was maintained in DMEM supplemented with 10% heat-inactivated FBS and antibiotics (100 U/mL of penicillin, 100 µg/mL of streptomycin) at 37 °C in humidified 5% CO_2_ and 95% air.

Peritoneal macrophages were isolated from 8 weeks old C57BL/6 and MyD88 knockout mice. Peritoneal macrophages were obtained by peritoneal lavage from mice that were injected with 2 mL of 4% thioglycollate broth (BD Diagnostic Systems; Sparks, MD, USA). Thioglycollate-elicited peritoneal macrophages were collected from peritoneal cavity of mice three days after injection.

### 3.3. Animals and LPS-Induced ALI Mouse Model

Female BALB/*c* mice (22–25 g, 8 weeks old) were purchased from Orient Bio (Seoul, Korea). C57BL/6 MyD88-deficient (MyD88^−/−^) mice were described [19]. They were housed in groups of 5 under standard conditions (temperature 22 ± 2 °C, humidity 55% ± 5%, 12-h light/dark cycle) with food and water *ad libitum*. The study protocol was reviewed and approved by the Committee on the Ethics of Animal Experiments of the Konkuk University (Permit Number: KU13102, 23 July 2013). The detailed protocol for LPS-induced ALI mouse model was described in previous report [20].

### 3.4. Real-Time Polymerase Chain Reaction (PCR)

Total RNA was isolated from cells or tissue using QIAzol lysis reagent (QIAGEN, Hilden, Germany) according to the manufacturer’s instructions. 1 mg of RNA was reversely transcribed to make cDNA using Maxima H Minus First Strand cDNA synthesis Kit (Thermo Scientific, Waltham, MA, USA).

Real-time PCR was performed using 2X SYBR Green Supermix (Bio-Rad, Hercules, CA, USA) with an iCycler (Bio-Rad) using each of the following primers: IL-1β (5'-AGCAATGACTCCAAAGTAGACC-3' and 5'-ATCTTTTGGGGTCCGTCAACT-3'), IL-6 (5'-ATCCAGTTGCCTTCTTGGGACTGA-3' and 5'-TTGGATGGTCTTGGTCCTTAGCCA-3'), IFN-β (5'-CAGCTCCAAGAAAGGACGAAC-3' and 5'-GGCAGTGTAACTCTTCTGCAT-3') and HPRT (5'-TCAGTCAACGGGGGACATAAA-3' and 5'-GGGGCTGTACTGCTTAACCAG-3'). The primers were purchased from Bioneer (Seoul, Korea). The PCR condition was 7 min at 95 °C, followed by 40 cycles of 95, 55 and 72 °C for 30 s. The resulting mean DCt values from each group were then used to calculate relative changes of mRNA expression as the ratio (R) of mRNA expression.

### 3.5. Hematoxylin and Eosin (H&E) Staining

Lung tissues were harvested 24 h after the injection of LPS, then fixed with 4% paraformaldehyde for 24 h, and embedded in paraffin medium and sectioned (4 µm). The tissue-sectioned samples were stained with H&E. Lung edema and inflammatory cell infiltration were evaluated by observation under light microscopy.

### 3.6. Other Experiments

The determination of nitrite concentration and experimental condition for enzyme-linked immunosorbent assay (ELISA) and immunoblot analysis were determined as described previously [19].

### 3.7. Statistical Analysis

The results were expressed as the mean ± standard error of the mean (SEM) of at least three independent experiments (*n* = 3). Statistical analysis was performed using the Student’s *t*-test, and *p* values less than 0.05 were considered to be statistically significant.

## 4. Conclusions

Streptochlorin strongly inhibited LPS-induced proinflammatory mediators such as NO, pro-IL-1β, and IL-6 in RAW264.7 cells through inhibition of the TRIF-dependent signaling pathway. Streptochlorin inhibited TRIF-dependent signaling from LPS-primed TLR4, leading to reduced activation of IRF3 and STAT1. Streptochlorin also attenuated LPS-induced ALI via suppression of neutrophil infiltration and proinflammatory cytokine production, such as TNF-α and IL-6.

## References

[1] Huang G.J., Pan C.H., Liu F.C., Wu T.S., Wu C.H. (2012). Anti-inflammatory effects of ethanolic extract of *Antrodia salmonea* in the lipopolysaccharide-stimulated RAW246.7 macrophages and the λ-carrageenan-induced paw edema model. Food Chem. Toxicol..

[2] Koppula S., Kim W., Jiang J., Shim D., Oh N., Kim T., Kang T., Lee K. (2013). Carpesium macrocephalum attenuates lipopolysaccharide-induced inflammation in macrophages by regulating the NF-κB/IκB-α, Akt, and STAT signaling pathways. Am. J. Chin. Med..

[3] Akira S., Uematsu S., Takeuchi O. (2006). Pathogen recognition and innate immunity. Cell.

[4] Covert M.W., Leung T.H., Gaston J.E., Baltimore D. (2005). Achieving stability of lipopolysaccharide-induced NF-κB activation. Science.

[5] Fitzgerald K.A., McWhirter S.M., Faia K.L., Rowe D.C., Latz E., Golenbock D.T., Coyle A.J., Liao S., Maniatis T. (2003). IKKepsilon and TBK1 are essential components of the IRF3 signaling pathway. Nat. Immunol..

[6] Bowie A.G., Haga I.R. (2005). The role of Toll-like receptors in the host response to viruses. Mol. Immunol..

[7] Perry A.K., Gang C., Zheng D., Hong T., Cheng G. (2005). The host Type I interferon response to viral and bacterial infections. Cell Res..

[8] Grommes J., Vijayan S., Drechsler M., Hartwig H., Mörgelin M., Dembinski R., Jacobs M., Koeppel T.A., Binnebösel M., Weber C. (2012). Simvastatin reduces endotoxin-induced acute lung injury by decreasing neutrophil recruitment and radical formation. PLoS ONE.

[9] Matute-Bello G., Frevert C.W., Martin T.R. (2008). Animal models of acute lung injury. Am. J. Physiol..

[10] Choi I.K., Shin H.J., Lee H.S., Kwon H.J. (2007). Streptochlorin, a marine natural product, inhibits NF-κB activation and suppresses angiogenesis *in vitro*. J. Microbiol. Biotechnol..

[11] Shin H.J., Jeong H.S., Lee H.S., Park S.K., Kim H.M., Kwon H.J. (2007). Isolation and structure determination of streptochlorin, an antiproliferative agent from a marine-derived *Streptomyces* sp. 04DH110. J. Microbiol. Biotechnol..

[12] Lee S., Shin H.J., Kim D., Shim D., Kim T., Ye S., Won H., Koppula S., Kang T., Lee K. (2013). Streptochlorin suppresses allergic dermatitis and mast cell activation via regulation of Lyn/Fyn and Syk signaling pathways in cellular and mouse models. PLoS ONE.

[13] Janeway C.A., Medzhitov R. (2002). Innate immune recognition. Annu. Rev. Immunol..

[14] Kawai T., Takeuchi O., Fujita T., Inoue J., Muhlradt P.F., Sato S., Hoshino K., Akira S. (2001). Lipopolysaccharide stimulates the MyD88-independent pathway and results in activation of IFN-regulatory factor 3 and the expression of a subset of lipopolysaccharide-inducible genes. J. Immunol..

[15] Toshchakov V., Jones B.W., Perera P., Thomas K., Cody M.J., Zhang S., Williams B.R., Major J., Hamilton T.A., Fenton M.J. (2002). TLR4, but not TLR2, mediates IFN-Β-induced STAT1α/Β-dependent gene expression in macrophages. Nat. Immunol..

[16] Samavati L., Rastogi R., Du W., Hüttemann M., Fite A., Franchi L. (2009). STAT3 tyrosine phosphorylation is critical for interleukin 1β and interleukin-6 production in response to lipopolysaccharide and live bacteria. Mol. Immunol..

[17] Bode J.G., Ehlting C., Häussinger D. (2012). The macrophage response towards LPS and its control through the p38^MAPK^–STAT3 axis. Cell Signal..

[18] Knapp S. (2010). LPS and bacterial lung inflammation models. Drug Discov. Today.

[19] Adachi O., Kawai T., Takeda K., Matsumoto M., Tsutsui H., Sakagami M., Nakanishi K., Akira S. (1998). Targeted disruption of the *MyD88* gene results in loss of IL-1- and IL-18-mediated function. Immunity.

[20] Shim D., Han J., Sun X., Jang C., Koppula S., Kim T., Kang T., Lee K. (2013). *Lysimachia clethroides* Duby extract attenuates inflammatory response in RAW264.7 macrophages stimulated with lipopolysaccharide and in acute lung injury mouse model. J. Ethnopharmacol..

